# Breastfeeding: Biological and Social Variables in Different Modes of Conception

**DOI:** 10.3390/life11020110

**Published:** 2021-02-01

**Authors:** Paola Pileri, Ilenia di Bartolo, Martina Ilaria Mazzocco, Giovanni Casazza, Sofia Giani, Irene Cetin, Valeria Maria Savasi

**Affiliations:** 1 Department of Woman, Child and Neonate, Buzzi Children Hospital, ASST Fatebenefratelli Sacco, via L. Castelvetro 32, 20154 Milan, Italy; ilenia.dibartolo@unimi.it (I.d.B.); martina.mazzocco@libero.it (M.I.M.); irene.cetin@unimi.it (I.C.); 2 Department of Biomedical and Clinical Sciences, Università degli Studi di Milano, via G.B. Grassi 74, 20157 Milan, Italy; giovanni.casazza@unimi.it (G.C.); sofigiani@gmail.com (S.G.); valeria.savasi@unimi.it (V.M.S.); 3 Department of Woman, Child and Neonate, Luigi Sacco Hospital, ASST Fatebenefratelli Sacco, via G.B. Grassi 74, 20157 Milan, Italy

**Keywords:** breastfeeding, assisted reproductive technology, mode of conception, ovum donation

## Abstract

Background: Breastfeeding has effects on health throughout the lives of mothers and babies. In 2014 in Italy, 10,976 babies were born through ART (assisted reproductive technology), accounting for 2.2% of annual births. The study aims to assess how both social and biological variables and the mode of conception influence breastfeeding. Methods: This observational study involves 161 pregnancies from three different modes of conception: homologous in vitro fertilization, ovum donation, and spontaneous pregnancies. Neonatal and maternal characteristics were collected from the hospital database, while breastfeeding outcomes were obtained through telephone interviews. Results: The mode of conception did not influence any of the breastfeeding outcomes. Breastfeeding duration was negatively affected by smoking. Vaginal delivery, birth weight > 2500 g, delivery > 37 gestational weeks, breastfeeding intention, and rooming-in are positively associated with the initiation of breastfeeding, while skin-to-skin contact and receiving information concerning breastfeeding are the most significant variables associated with its exclusivity and duration. Conclusions: The duration and exclusivity of breastfeeding are mainly related with information thereon, promotion, and breastfeeding support, but not with the mode of conception. It is essential to adequately support women from the outset in breastfeeding, as recommended by the World Health Organization (WHO) guidelines.

## 1. Introduction

Breastfeeding has positive effects on mothers and their breastfed babies, enduring throughout life [1]. It is the earliest form of communication between mother and child and it is species-specific. Breast milk is the best food for infants, as it provides all the nutrients needed in the first phase of life and contains bioactive and immunological substances that are not found in artificial substitutes. It prevents the development of diseases, both in the short term and in the long term [2,3]. Moreover, breastfeeding stimulates the natural uterine contractions, reducing post-partum bleeding, and is associated with a lower risk of breast cancer [1,2,3,4]. These are the reasons why the World Health Organization (WHO) [5] recommends exclusive breastfeeding for the first 6 months of the infant’s life, and continued breastfeeding up to 2 years and beyond. The prevalence of breastfeeding is not homogeneous among different countries [6]. In 2006–2012, only 25% of children in Europe and 43% in South-East Asia were exclusively breastfed for their first 6 months of life [7]. Breastfeeding promotion and all the strategies of breastfeeding support gave positive results in terms of improved breastfeeding initiation rates, but further efforts are needed to achieve satisfactory results, above all with regard to patients undergoing assisted reproductive technology (ART), which are increasing and on which little data are available.

In 2014, ART treatments enabled to deliver 11,272 babies, accounting for 2.2% of the total number of infants born in that year [8]. As regards the heterologous in vitro fecundation techniques, there are significant increases in the number of treated couples (from 2462 in 2015 to 5450 in 2016) but also in the number of live births (from 601 in 2015 to 1457 in 2016) [9]. Women who conceive with in vitro fertilization (IVF) are more susceptible to pregnancy complications due to the fact that they are usually older than women who conceive spontaneously [10,11]. There are few studies on the effects of infertility treatments on breastfeeding outcomes, as there is a variety of potential biases related to the choice of the right comparison groups or of the appropriate sources of recruitment. We designed an observational study to investigate the biological, clinical, and social variables as well as the influence of the mode of conception on breastfeeding. Moreover, we investigated the initiation, continuation, and exclusivity of breastfeeding in two groups of women who had conceived through IVF techniques, compared with a matched control group of mothers who had conceived spontaneously.

## 2. Materials and Methods

**Figure 1:**
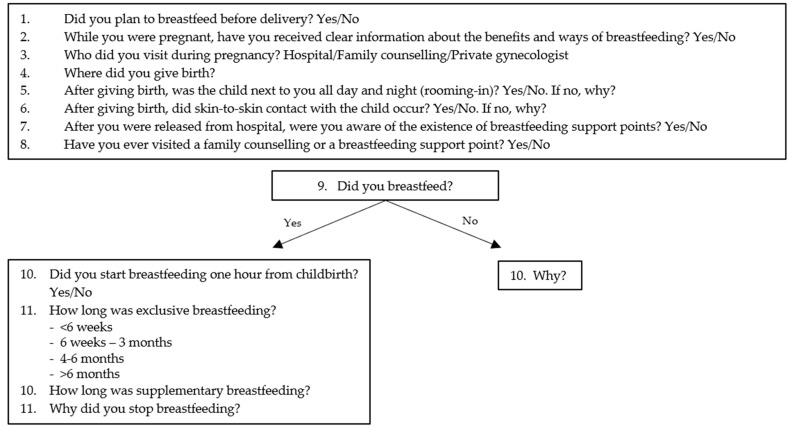
Questionnaire used to obtain information on breastfeeding outcomes by means of telephone interviews.

This study was observational, and all data were recorded in an anonymous way, so that the individuals were not identifiable, meaning it is exempted from ethical approval and permission. It was conducted in the Fertility center and in the Obstetrics and Gynecology Department of the Luigi Sacco Hospital at the University of Milan, situated northwest of Milan, in Lombardy, where about 80,000 women live and low socio-economic groups are prevalent. Milan is a UNICEF (United Nations Children’s Fund) Baby Friendly Community and L. Sacco Hospital is a University Hospital without neonatal intensive care, with an average of 1200 deliveries a year in the period studied. One hundred and sixty-one pregnant women who delivered between January 2014 and March 2016 were enrolled and stratified based on three different modes of conception. We used the definition of homologous in vitro fertilization, including homologous IVF and embryo transfer (IVF-ET) or intracytoplasmatic sperm injection (ICSI). As regards heterologous fertilization, we used the definition ovum donation (OD) for pregnancies obtained by egg donation—no pregnancy was obtained by sperm donation. We enrolled 45 participants in the IVF group, 26 participants in the OD group and 90 pregnancies conceived spontaneously. We selected pregnancies with gestational age at delivery greater than 37 weeks, excluding multiple pregnancies, neonatal genetic chromosomal abnormalities, and maternal diseases, such as HIV (Human Immunodeficiency Virus), for which breastfeeding is contraindicated. Maternal variables defined at admission were age, nationality, marital status, education level, occupation, pregestational body mass index (BMI), weight gain during pregnancy, and smoking. Obstetric and neonatal variables defined at birth were parity, delivery mode, pain medication during delivery, gestational week, birthweight, and Apgar score at 5 min < 7. Breastfeeding outcomes were assessed using WHO-recommended definitions [12]: exclusive breastfeeding means that the infant receives only breast milk (including expressed breast milk) and, possibly, drops, syrups (vitamins, minerals, drugs), but nothing else, predominant breastfeeding means that the infant receives mostly breast milk, but also takes other non-nutritive liquids (e.g., water, glucose solution), and complementary breastfeeding means that the infant receives both breast milk and non-human milk (food, liquid, and formula). The good medical practices included some of the “ten steps to successful breastfeeding” (WHO-UNICEF) [13]: information about breastfeeding (step 3), skin-to-skin contact (step 4), rooming-in (enable mothers and their infants to remain together 24 h a day) (step 7), and information about the existence of breastfeeding support points and post-natal support (step 10). We used our medical records and neonatal database to collect all the anthropometric neonatal data, data on the mode of delivery, socio-demographic, and lifestyle characteristics. We obtained information on breastfeeding outcomes by means of telephone interviews carried out after six months from the birth. We assessed the initiation, duration, and exclusivity of breastfeeding by means of fixed-choice retrospective questions (see questionnaire in Figure 1). Continuous variables were reported as mean (standard deviation (SD)) and categorical variables were reported as counts (percentage). Maternal, obstetric, neonatal characteristics, and good medical practices were analyzed separately to assess their influence on the mode of conception. Chi-square or Fisher exact test, as appropriate, were used to assess the association with the mode of conception and the abovementioned characteristics. In addition, a logistic regression analysis was performed to assess the effect of the same maternal, obstetric, and neonatal variables and good medical practices on initiation, duration, and exclusivity of breastfeeding. Univariate and multivariate logistic regression analyses were performed. At the multivariate stage, only the variables that were statistically significant at the univariate step were considered. Three separate analyses were conducted for the three breastfeeding outcomes. Odds ratios (OR), with their 95% confidence intervals (CI), were obtained from logistic models. A *p*-value < 0.05, two-sided, was considered statistically significant. All statistical analyses were conducted using SAS software (release 9.4) (SAS Italy, Milan, Italy).

## 3. Results

The characteristics of our sample are shown in Table 1 and Table 2. Most of the women with spontaneous pregnancy were multiparous (56.6%), whereas IVF and OD were primiparous (91.1% and 73.08%, *p* < 0.005, respectively). A significantly high rate of OD pregnancies were delivered with scheduled cesarean section. Small for Gestational Age (SGA) infants (birthweight < 2500 g) were significantly more frequent in the IVF group (17.78% vs. 3.33% in spontaneous pregnancies vs. 0% in ovum donation pregnancies, *p* < 0.005). We found statistically significant differences in rooming-in and skin-to-skin practices among the three groups. These practices are more frequent in spontaneous pregnancies (88.8% and 82.22%) than in the IVF group (75.5% and 64.4%) and OD group (69.23% and 61.54%). 

The initiation, duration, and exclusivity of breastfeeding were not affected by the mode of conception, as shown in Table 3. Of women who had a spontaneous pregnancy, 85 (94.44%) wanted to breastfeed, 80 started breastfeeding (88.89%), and 61 of these (76.25%) breastfed for longer than 6 months, 55 (68.75%) with exclusive breastfeeding. Of women who had conceived through IVF, 41 (91.11%) wanted to breastfeed, 39 (86.67%) started breastfeeding, and 22 of these (56.41%) continued for more than 6 months, 21 (53.85%) exclusively. Of the women who had conceived with ovum donation, 22 (84.62%) had the intention to breastfeed, 19 (73.08%) breastfed, 13 of these (68.42%) for longer than 6 months, and 9 (47.37%) with exclusive breastfeeding. Maternal choice was the most frequent reason linked to breastfeeding failure in spontaneous conception, while insufficient milk supply was more frequent in IVF and OD groups. Maternal breast diseases were the most common reason for breastfeeding failure (before 6 months) in all groups. 

Data showed that smoking was the unique maternal variable that reduced the duration of breastfeeding (OR 6.3, 95% CI 1.18–34.19). The initiation of breastfeeding was associated with vaginal delivery at term (OR 5.7, *p* < 0.05) and birthweight over 2500 g (OR 6.11, *p* < 0.05). Multiparity and delivery at term positively influenced the continuation of breastfeeding (OR 3.07, *p* < 0.05 and OR 1.58, *p* < 0.005, respectively). Exclusive breastfeeding practices were favored by multiparity and vaginal delivery (OR 3.3, *p* < 0.005 and OR 5.45, *p* < 0.05, respectively) (Table 4). 

Breastfeeding intention and rooming-in are two variables positively related with the breastfeeding initiation, and rooming-in positively influenced breastfeeding exclusivity (OR 2.97, *p* < 0.05). Moreover, skin-to-skin contact and awareness about all the benefits of breastfeeding, as well as about breastfeeding support centers, were the most significant variables associated with duration (OR 2.49, *p* < 0.05, OR 2.99, *p* < 0.05, and OR 2.80, *p* < 0.05, respectively) and exclusive breastfeeding (skin-to-skin OR 4.66, *p* < 0.005, information about breastfeeding support OR 2.69, *p* < 0.05) (Table 5).

## 4. Discussion

Our study was the first to compare the different modes of conception with breastfeeding outcomes. Most of the participants started breastfeeding regardless of the conception mode (88% in spontaneous, 86% in IVF, and 73% in OD group, *p* = 0.12), and a large portion of them continued for longer than 6 months (76%, 56%, 68%, *p* = 0.1691, respectively). Similarly, in a large Canadian retrospective cohort study involving 76 women who had conceived through ART, no significant differences were found between women who had conceived spontaneously and those who had conceived through ART [14]. That study had certain limitations. More specifically, given that it involved cases and control group subjects coming from the same source population, it did not consider all the factors which could interfere with breastfeeding outcomes, such as the mode of delivery, the hospital where women delivered, and the gestational week of delivery; therefore, the collected data could have been biased. On the contrary, an Australian prospective cohort study by Hammarberg et al. [15], involving 183 ART pregnancies, showed a declined proportion of breast milk amounting to 77% in ART participants by 6 weeks, claiming that anxiety during pregnancy was the most important reason for early cessation of breastfeeding in the group of ART pregnancies. However, the two considered groups were not homogenous, i.e., they differed in many aspects such as education, parity, mode of delivery, age, and neonatal weight. This might have contributed to the result. Even a recent retrospective Italian study, including 173 singletons who had conceived with the help of ART, reported a greater proportion of breastfeeding cessation in that group of patients at 6 post-partum weeks [16], suggesting a wide range of reasons to be taken into account, including socio- demographics and obstetric variables. Interestingly, we studied mothers who had conceived with ovum donation too, and none of them ceased to breastfeed after 6 weeks. We also investigated the reasons for not initiating breastfeeding or for early cessation of breastfeeding in women: 66% of mothers who had conceived spontaneously did not breastfeed for choice as first reason, whereas insufficient milk production was the most frequently reported reason in IVF and OD mothers (66% and 42%, respectively). This result was not explained by the mammary gland inability or by hormonal differences due to the mode of conception, because we showed that when mothers started to breastfeed, they continued for 6 months in 56.41% of the homologous IVF group and in 68.42% of the OD group. The insufficient milk supply could be influenced by the significant low rate of rooming-in and skin-to-skin contact in IVF and OD groups (skin to-skin 64.4% and 61.5% vs. 82.2%, *p* < 0.05, rooming in 69.2% and 75.5% vs. 88.9%, *p* < 0.05, respectively). It is known that the intimate contact evokes neurobehaviors ensuring achievement of basic biological needs and that it helps mothers to trust in their ability to breastfeed in the right way, thus promoting mother–child bonding and increasing the production of milk [17]. The lack of this good medical practice, due to the higher proportion of pregnancy complications in women who conceive through ART, such as operative delivery, premature birth, and labor induction, compared to women who conceive spontaneously, could explain why in our study, IVF and OD groups breastfed fewer than the group of spontaneous pregnancies and why they more frequently had an insufficient milk production. We showed that skin-to-skin contact was directly involved in the duration (OR 2.49, *p* < 0.05) and exclusivity of breastfeeding (OR 4.66, *p* < 0.005). Likewise, rooming-in was involved in the initiation (OR 2.97, *p* < 0.05) and exclusivity (OR 3.13, *p* < 0.05) of breastfeeding. The most important factor impacting significantly on the initiation was breastfeeding intention (OR 21.54, *p* < 0.001). Breastfeeding counseling therefore plays an essential role during pregnancy. The most frequently reported reason for breastfeeding failure (before 6 months) was a maternal disease for all groups. Maternal diseases leading to early breastfeeding discontinuation are mastitis, abscesses, and yeast infections, as reported in References [18,19]. Our study found that not all women received clear information about breastfeeding benefits during pregnancy. Interestingly, the mothers in the group of ovum donation were more informed than the others (96.15% vs. 80% in the homologous IVF group and 92.22% in the spontaneous group). Despite this, fewer women who had conceived with ovum donation wanted to breastfeed before delivery compared to the others, although not significantly. We also showed that women who had conceived through homologous IVF received less information about breastfeeding and neonatal assistance (69.89% vs. 80.77% of the ovum donation group and 88.89% of the spontaneous pregnancy group). In fact, only 56.41% of them kept on breastfeeding after 6 months from delivery. This result should lead to provide more information on the benefits of breastfeeding during pregnancy in women who achieved pregnancy by IVF techniques, as promoted by the step 3 of the “ten steps to successful breastfeeding” (WHO-UNICEF) [13]. In addition, our study takes into account the maternal and neonatal variables related to the initiation, continuation, and exclusivity of breastfeeding. As we showed, smokers are more likely to stop breastfeeding before 6 months. This data was in line with an Australian longitudinal study that reported a shorter duration of breastfeeding in smoking mothers than non-smoking mothers [20]. Our results confirmed that parity impacts on breastfeeding duration and exclusivity (multiparity versus primiparity OR 3.07, *p* < 0.05 and OR 3.3, *p* < 0.005, respectively). Hackman et al. [21] showed that women who had a previous experience of breastfeeding were more likely to breastfeed throughout 6 months, but they did not investigate exclusivity. Most of our IVF and OD participants were primiparous (91% and 73%). Notwithstanding this, the mode of conception did not influence the breastfeeding features, probably because the mothers understood the potential difficulties related to breastfeeding. The mode of delivery influenced breastfeeding, especially when comparing vaginal delivery to urgent cesarean section: vaginal delivery was significantly associated with initiation and exclusivity of breastfeeding (OR 5.7, *p* < 0.05 and OR 5.45, *p* < 0.05). Similarly, a recent prospective pregnancy cohort study found that urgent cesarean sections implied a higher proportion of breastfeeding difficulties [22]. In our sample, women who had conceived with OD often had an urgent cesarean section (61.5%), and this could have influenced the significantly low rate of skin-to-skin contact (61.5%, *p* < 0.05). As our data showed, breastfeeding intention is the most significant factor influencing breastfeeding initiation and duration. Women who wanted to breastfeed did so for longer than the others.

One of the limitations of our study is that the use of telephone interviews to collect most of the data could have introduced possible biases. Moreover, we carried out this study in a single hospital complex, so the generalizability of the findings may be limited. Lastly, considering maternal characteristics and obstetric factors as variables to match could lead to confounding factors.

## 5. Conclusions

In our study, we found that breastfeeding support and promotion are the most significant factors which could affect breastfeeding outcomes. Ovum donation does not negatively impact on breastfeeding. The high percentage of failure and early cessation of breastfeeding due to low supply of milk and to maternal diseases suggest the need for a great support from qualified staff, as recommended by WHO guidelines.

## Figures and Tables

**Table 1:** Population characteristics: socio-economic data.

	SpontaneousN = 90	IVF N = 45	OD N = 26	*p*-Value *
Maternal age years	35.02 ± 2.98	35.61 ± 4.47	43.50 ± 3.99	
Pre-pregnancy BMI, kg/m^2^	22.46 ± 3.66	21.98 ± 3.14	20.70 ± 2.42	
Weight gain in pregnancy, kg	11.97 ± 4.65	14.22 ± 3.60	9.27 ± 3.92	
Obesity, n (%)	5 (5.56)	1 (2.22)	0 (0.00)	0.6119
Smokers, n (%)	3 (3.33)	5 (11.11)	1 (3.85)	0.1823
Married, n (%)	55 (61.11)	44 (97.78) **	11 (42.31)	<0.0001
Italian, n (%)	83 (92.22)	38 (84.44)	26 (100.00)	0.0795
Level of Education				0.2527
Bachelor’s degree n (%)	45 (50.00)	22 (48.89)	18 (69.23)	
Lower secondary school diploma, n (%)	35 (38.89)	17 (37.78)	8 (30.77)	
High secondary school diploma, n (%)	10 (11.11)	6 (13.33)	0 (0.00)	
Employed, n (%)	77 (85.56)	35 (77.78)	24 (92.31)	0.2784

Note: Values are n (%), unless otherwise indicated. * *p* < 0.05, ** *p* < 0.005.

**Table 2:** Population characteristics: medical data.

	SpontaneousN = 90	IVF N = 45	OD N = 26	*p*-Value *
Primiparous	39 (43.33)	41 (91.11) **	19 (73.08)	<0.0001
Mode of delivery				
Elective cesarean section	25 (27.78)	13 (28.89)	16 (61.54)	0.0014
Urgent cesarean section	10 (11.11)	7 (15.56)	4 (15.38)	
Vaginal spontaneous	50 (55.56)	24 (53.33)	3 (11.54)	
Vacuum	5 (5.56)	1 (2.22)	3 (11.54)	
Pain medication during delivery				0.0024
General anesthesia	1 (1.11)	3 (6.67)	0 (0.00)	
Spinal/epidural	58 (64.44)	29 (64.44)	25 (96.15)	
No pain medications	31 (34.44)	13 (28.89)	1 (3.85)	
Birthweight (g)	3263.48 ± 438.00	3022.11 ± 649.95	3314.19 ± 396.26	
Birthweight <2500 g	3 (3.33)	8 (17.78) **	0 (0.00)	0.0038
Gestational age (weeks)	39.07 ± 1.25	38.36 ± 2.44	38.77 ± 1.21	
Skin-to-skin	74 (82.22)	29 (64.44)	16 (61.54) *	
Rooming-in	80 (88.89)	34 (75.56)	18 (69.23) *	

Note: Values are n (%), unless otherwise indicated. * *p* < 0.05, ** *p* < 0.005.

**Table 3:** The relationship between mode of conception and breastfeeding.

Breastfeeding Outcomes	Spontaneous N = 90	IVF N = 45	OD N = 26	*p*-Value *
Intention to breastfeed	85 (94.44)	41 (91.11)	22 (84.62)	0.2469
Initiation of breastfeeding	80 (88.89)	39 (86.67)	19 (73.08)	0.1297
Duration of breastfeeding (in women who started breastfeeding)	n = 80	n = 39	n = 19	0.1691
<6 weeks	3 (3.75)	1 (2.56)	0 (0.00)	
6 weeks–3 months	9 (11.25)	8 (20.51)	5 (26.32)	
4–6 months	7 (8.75)	8 (20.51)	1 (5.26)	
>6 months	61 (76.25)	22 (56.41)	13 (68.42)	
Exclusive breastfeeding at 5–6 months	55 (68.75)	21 (53.85)	9 (47.37)	0.1070
Reason for not starting breastfeeding (in women who did not start)	n = 10	n = 6	n = 7	
Choice	6 (60.00)	1 (16.67)	2 (28.57)	
Milk supply	2 (20.00)	4 (66.67)	3 (42.86)	
Social problems	0 (0.00)	0 (0.00)	1 (14.29)	
Breast attack problems (e.g., breast pain during suction, fissures, malnutrition of the newborn)	2 (20.00)	0 (0.00)	1 (14.29)	
Neonatal diseases	0 (0.00)	1 (16.67)	0 (0.00)	
Reason for early cessation of breastfeeding before 6 months	n = 19	n = 17	n = 6	
Social problems	5 (26.32)	3 (17.65)	1 (16.67)	
Breast attack problems (e.g., breast pain during suction, fissures, malnutrition of the newborn)	4 (21.05)	2 (11.76)	0 (0.00)	
Maternal breast diseases	10 (52.63)	12 (70.59)	5 (83.33)	

Note: Values are n (%), unless otherwise indicated. * *p* < 0.05.

**Table 4:** Associations between maternal and perinatal variables and the initiation, continuation, and exclusivity of breastfeeding.

Maternal and Perinatal Characteristics	Breastfeeding		
	Initiation OR (95% CI)	Continuation OR (95% CI)	Exclusivity OR 95% CI
Age	0.94 (0.85–1.02)	1.015 (0.03–0.13)	0.94 (0.87–1.01)
Nationality Italian vs. Foreign	0.44 (0.05–3.51)	0.66 (0.17–2.53)	1.0 (0.31–3.24)
Obesity (BMI > 30) No vs. Yes	1.21 (0.13–10.84)	1.55 (0.24–9.63)	1.07 (0.17–6.63)
Smoking No vs. Yes	1.78 (0.34–9.16)	6.35 (1.18–34.19) *	2.23 (0.47–10.38)
Education			
High vs. lower secondary school	1.89 (0.49–7.17)	2.64 (0.72–9.57)	0.87 (0.24–3.10)
Degree vs. lower secondary school	2.50 (0.67–9.26)	2.41 (0.70–8.29)	1.43 (0.41–4.95)
Marital Status Maiden vs. Married	0.85 (0.33–2.15)	1.41 (0.62–3.16)	1.24 (0.58–2.63)
OccupationUnemployed vs. Employed	0.45 (0.15–1.29)	1.26 (0.42–3.76)	1.08 (0.39–2.94)
Mode of conception			
Spontaneous vs. Ovum donation pregnancies	2.95 (0.99–8.74)	1.48 (0.49–4.43)	2.44 (0.88–6.75)
Homologous IVF vs. Ovum donation pregnancies	2.39 (0.70–8.11)	0.6 (0.18–1.89)	1.3 (0.43–3.88)
Parity Multiparous vs. Primiparous	2.53 (0.88–7.21)	3.07 (1.35–6.93) *	3.3 (1.54–7.03) **
Mode of delivery			
Vacuum vs. Urgent CS ^a^	0.62 (0.11–3.46)	2.27 (0.20–24.88)	2.2 (0.323–14.97)
Vaginal spontaneous vs. Urgent CS ^a^	5.7 (1.37–23.63) *	1.29 (0.39–4.20)	5.45 (1.68–17.58) *
Scheduled CS vs. Urgent CS ^a^	1.22 (0.36–4.06)	0.69 (0.20–2.35)	3.05 (0.90–10.33)
Birthweight > 2500 g	6.11 (1.69–22.08) *	2.384 (0.46–12.33)	0.79 (0.14–4.49)
Gestational age > 37 weeks	1.28 (1.01–1.62) *	1.58 (1.16–2.14) **	1.27 (0.98–1.63)

Note: Values are n (%), unless otherwise indicated. * *p* < 0.05, ** *p* < 0.005, ^a^ CS: cesarean section.

**Table 5:** Influence of good medical practices on the initiation, continuation, and exclusivity of breastfeeding.

Good Medical Practices	Breastfeeding		
	Initiation OR (95% CI)	Continuation OR (95% CI)	Exclusivity OR 95% CI
Intention to breastfeed Yes vs. No	21.54 (5.86–79.02) ***	7.30 (0.73–72.42)	1.63 (0.22–11.91)
Provision of information about breastfeeding benefits Yes vs. No	0.78 (0.16–3.66)	2.99 (1.01–8.88) *	1.46 (0.49–4.30)
Rooming in Yes vs. No	2.97 (1.12–7.88) *	1.91 (0.73–4.95)	3.13 (1.19–8.16) *
Skin-to-skin Yes vs. No	1.29 (0.48–3.38)	2.49 (1.12–5.55) *	4.66 (2.05–10.55) **
Provision of information about the existence of breastfeeding support points Yes vs. No	1.32 (0.45–3.90)	2.80 (1.13–6.90) *	2.69 (1.09–6.61) *
Post-natal support Yes vs. No	1.46 (0.51–4.22)	0.87 (0.39–1.92)	0.91 (0.42–1.93)

Note: Values are n (%), unless otherwise indicated. * *p* < 0.05, ** *p* < 0.005, *** *p* < 0.001.

## Data Availability

The data presented in this study are available on request from the corresponding author. The data are not publicly available due to privacy.
